# Health-related quality of life in children with inflammatory brain disease

**DOI:** 10.1186/s12969-018-0291-4

**Published:** 2018-11-20

**Authors:** Elina Liu, Marinka Twilt, Pascal N. Tyrrell, Anastasia Dropol, Shehla Sheikh, Mark Gorman, Susan Kim, David A. Cabral, Rob Forsyth, Heather Van Mater, Suzanne Li, Adam M. Huber, Elizabeth Stringer, Eyal Muscal, Dawn Wahezi, Mary Toth, Pavla Dolezalova, Katerina Kobrova, Goran Ristic, Susanne M. Benseler

**Affiliations:** 1 Rheumatology, Department of Pediatrics, Alberta Children’s Hospital, 2888 Shaganappi Trail NW, Calgary, AB T3B 6A8 Canada; 2 School of Medicine, Queen’s University, Kingston, ON Canada; 3 Cumming School of Medicine, University of Calgary, Calgary, AB Canada; 4 Alberta Children’s Hospital Research Institute, Calgary, AB Canada; 5 Department of Medical Imaging and Department of Statistical Sciences, University of Toronto, Toronto, ON Canada; 6 Department of Rheumatology, Hospital for Sick Children, Toronto, ON Canada; 7 Boston’s Children Hospital, Boston, MA USA; 8 Benioff Children’s Hospital, University of California, San Francisco, California USA; 9 BC Children’s Hospital, Vancouver, BC Canada; 10 Institute of Neuroscience, Newcastle University, Newcastle upon Tyne, UK; 11 Duke Children’s Hospital & Health Centre, Durham, North Carolina USA; 12 Joseph M. Sanzari Children’s Hospital, Hackensack, NJ USA; 13 IWK Health Centre and Dalhousie University, Halifax, NS Canada; 14 Texas Children’s Hospital, Houston, TX USA; 15 Children’s Hospital at Montefiore, Bronx, New York USA; 16 Akron Children’s Hospital, Akron, OH USA; 17 Charles University in Prague, Prague, Czech Republic; 18 Mother and Child Health Care Institute of Serbia, Belgrade, Serbia

**Keywords:** Pediatrics, Inflammatory brain disease, CNS vasculitis, Health-related quality of life, Quality of life

## Abstract

**Objective:**

To quantify the impact of inflammatory brain diseases in the pediatric population on health-related quality of life, including the subdomains of physical, emotional, school and social functioning.

**Methods:**

This was a multicenter, observational cohort study of children (< 18 years of age) diagnosed with inflammatory brain disease (IBrainD). Patients were included if they had completed at least one Health Related Quality of Life Questionnaire (HRQoL). HRQoL was measured using the Pediatric Quality of Life Inventory Version 4.0 (PedsQL) Generic Core Scales, which provided a total score out of 100. Analyses of trends were performed using linear regression models adjusted for repeated measures over time.

**Results:**

In this study, 145 patients were included of which 80 (55%) were females. Cognitive dysfunction was the most common presenting symptoms (63%), and small vessel childhood primary angiitis of the CNS was the most common diagnosis (33%). The mean child’s self-reported PedsQL total score at diagnosis was 68.4, and the mean parent’s proxy-reported PedsQL score was 63.4 at diagnosis. Child’s self-reported PedsQL scores reflected poor HRQoL in 52.9% of patients at diagnosis. Seizures or cognitive dysfunction at presentation was associated with statistically significant deficits in HRQoL.

**Conclusion:**

Pediatric IBrainD is associated with significantly diminished health-related quality of life. Future research should elucidate why these deficits occur and interventions should focus on improving HRQoL in the most affected subdomains, in particular for children presenting with seizures and cognitive dysfunction.

## Introduction

Childhood inflammatory brain diseases (IBrainDs) encompass a group of devastating conditions with high disease burden presenting with substantial neurological dysfunction in previously healthy children [1–4]. IBrainDs result from immune system activation against structures within the central nervous system (CNS), including both the brain and spinal cord, resulting in inflammation. Types of IBrainD affecting children include small vessel childhood primary angiitis of the CNS (SV-cPACNS), anti-N-methyl-D-aspartate (NMDA) receptor encephalitis, multiple sclerosis (MS), and Rasmussen’s encephalitis. These conditions often present acutely with one or more neurological symptoms, including cognitive and behavioural dysfunction, seizure, hallucination and hemiparesis [1, 4–7]. Systematic use of immunosuppressive therapy has improved survival and reduced long-term neurological deficits in childhood IBrainD, though significant burden of illness still exists [5].

Health-related quality of life (HRQoL) is a concept which considers the multidimensional contributions of physical, emotional and social functioning when defining wellbeing [8]. In pediatric populations especially, HRQoL has become an important central outcome, requiring separate evaluation from traditional medical definitions of outcome such as mortality [8–10]. It is increasingly used in both drug trials, and the clinical setting, to guide treatment and rehabilitation decisions [8, 9]. The Pediatric Quality of Life Inventory Version 4.0 Generic Core Scales (PedsQL) is a validated questionnaire that assesses pediatric HRQoL under four subdomains of functioning: physical, emotional, social and school [9]. Thus, poor PedsQL scores can reflect outcomes such as depression, fatigue, poor school participation, and social strains, which are difficult to quantify using other outcome scales but are critical considerations for assessing the long-term burden of IBrainD.

HRQoL in children with IBrainD has not yet been systematically evaluated. Although there is a perceived high disease burden for children with IBrainD and their families, there is limited existing literature. The impact of IBrainD on HRQoL, the clinical phenotypes associated with poor HRQoL, the subdomains of functioning most affected in IBrainD, and the relationship between early and late measures of HRQoL are not currently known.

Therefore, the aims of the study were to [1] describe the characteristics of patients with IBrainD, including clinical phenotype and HRQoL, [2] compare both the burden of IBrainD on the different subdomains within HRQoL, and patient perspectives of HRQoL against parent/proxy perspectives in children with IBrainD and [3] identify trajectories and risk factors for impaired HRQoL.

## Patients and methods

### Patients

This BrainWorks study was a multicenter, observational cohort study of children diagnosed with IBrainD between August 2001 and August 2016. BrainWorks is an international multicenter collaborative study aimed at increasing recognition, promoting rapid diagnostic evaluation and optimizing treatment for pediatric IBrainD. For new patients enrolled in BrainWorks, measurement of outcomes occurred at pre-specified intervals, including diagnosis. Patients from the BrainWorks study were included if they met diagnostic criteria for an IBrainD as defined by the treating physician at 18 years of age or younger, and had completed at least one HRQoL Questionnaire.

### Demographic and clinical features

Age at diagnosis, gender, and time to diagnosis were evaluated. Diagnosis was categorized into small vessel childhood primary angiitis of the CNS (SV-cPACNS), angiography-positive non-progressive childhood primary angiitis of the CNS (APNP-cPACNS), angiography-positive progressive childhood primary angiitis of the CNS (APP-cPACNS), antibody-mediated IBrainD, demyelinating IBrainD, granulomatous IBrainD, Moyamoya phenotype, Secondary CNS vasculitis, T-cell mediated IBrainD, and unclassified IBrainD. Anti-NMDA receptor encephalitis, Hashimoto’s encephalopathy, anti-glutamic acid decarboxylase antibody encephalitis, mycoplasma-associated encephalitis and neuromyelitis optica were categorized under antibody-mediated IBrainD.

### Disease activity, damage and neurological functioning

Neurological clinical features at time-of-diagnosis collected to the BrainWorks database included presence of seizures (focal, generalized, or status epilepticus), hemiparesis and cognitive dysfunction. Cognitive dysfunction included memory loss and behavioural abnormality.

Estimated disease activity and estimated damage were measured as separate continuous variables using the physician global assessment (PGA). Disease activity is determined based on the presence of symptoms and positive markers of disease activity, whereas estimated damage is an estimate of permanent structural damage and functional impairment. Both scores were reported by physicians on a visual analog scale out of 10 cm, where 0 cm represented no disease activity/estimated damage and 10 cm represented high disease activity/estimated damage.

Neurological functioning was characterized using the pediatric stroke outcome measure (PSOM), which assesses neurological and functional deficit across 5 subscales: right sensorimotor, left sensorimotor, language production, language comprehension and cognitive/behavioural deficit [11]. Overall neurological deficit in the PSOM is scaled out of 10, with each subscale measured out of 2. Higher values represent greater deficit. PSOM score at 12 months was assessed as a discrete variable, with any subscale score greater than 0.5 indicating the presence of neurological dysfunction.

### Health-related quality of life (HRQoL)

HRQoL was captured using the PedsQL, a validated tool built of two 23-question surveys; one self-report for pediatric patient completion, and one proxy-report for completion by the patient’s parent or caregiver [12]. Both PedsQL questionnaires were routinely completed at time-of-diagnosis and at each follow-up clinical visit, clinical situation permitting. Total PedsQL score was calculated for each report by taking the sum-total average of item scores across the four subdomains [9]. PedsQL scores were reported on a 0–100 scale, with higher scores representing superior HRQoL.

The four subdomains of PedsQL are physical (8 items), emotional (5 items), social (5 items) and school (5 items) functioning. A score can be reported for each subdomain by taking a sum-total average of the individual item scores within the subdomain. Alternatively, many sources often report only two subdomain scores: physical functioning and psychosocial functioning, where the psychosocial subdomain is a sum-total average of the item scores from the emotional, social and school functioning sections of the questionnaire. All subdomain PedsQL scores are also reported on a 0–100 scale, with higher scores representing superior HRQoL.

Poor HRQoL was defined as a child’s self-reported PedsQL score of ≤70, or a parent-proxy reported PedsQL score ≤ 65. Various studies have established PedsQL cut-off scores to differentiate good quality of life from notable impairment in quality of life, with the most frequent cut-off scores reported to be in the range of 70–79 [9, 13–15]. The cut-off scores for this study were chosen based on a study by Kim et al., which concluded that a PedsQL cut-off score of 70 separated patients with low symptoms and high quality of life from those with moderate-to-severe symptoms, and a study by Varni et al., which used PedsQL child self-reported total scores of 69.1 and a PedsQL parent proxy-reported total scores of 65.4 as “meaningful cut-off point(s) for impaired HRQoL”, at one standard deviation below the mean PedsQL score for healthy children [9, 14].

### Outcomes

The primary outcome assessed was the pediatric HRQoL, defined by PedsQL total score. Secondary outcomes were the HRQoL subdomains of physical, emotional, social and school functioning.

### Statistical analysis

Descriptive statistics for demographic and clinical variables are reported as means and standard deviation or medians with interquartile range for continuous variables and frequencies/proportions for categorical variables. Univariable linear regression models with a maximum likelihood algorithm for parameter estimation were used to test the association between PedsQL scale and summary scores with age at diagnosis, time to diagnosis, and estimated damage at diagnosis as well as the association between gender and the presence of hemiparesis, cognitive/behavioural dysfunction, or seizures at diagnosis and PedsQL scores at diagnosis. Regression models were adjusted for repeated measures with an autoregressive covariance structure in order to establish whether these relationships changed statistically significantly with time (log transformed). Parameter estimate and its standard error are reported, where parameter estimate represents the change ratio for each increase of 1 unit in the independent variable (unless otherwise indicated). Parameter estimates were used to estimate the difference between PedsQL scores of patients with different presentations at time of diagnosis to account for the longitudinal analysis and the variable PedsQL questionnaire completion. All statistical test results were considered significant at the *P* < .05 level. SAS 9.4 for Windows (SAS Institute Inc., Cary, NC, USA) was used for all analyses.

## Results

### Patients

A total of 145 patients from 13 international sites were included in the study; 80 (55%) female and 65 (45%) male; median age at diagnosis 10.3 years (range = 0.4–18.2 years); median time to diagnosis 1.2 months (range = 0–120 months); median follow-up of 24 months (range = 0–160 months) in the proxy-reports, and 28 months (range = 0–160 months) in the self-reports. The most common diagnoses were SV-cPACNS (48 children, 33%), antibody-mediated IBrainD (28 children, 18%), and APNP-cPACNS (22 children, 15%). One patient died. Patient demographic data are summarized in Table 1.

**Table 1:** Patient demographics for 145 pediatric patients with inflammatory brain disease

Female. Number (%)	80 (55%)
Age at diagnosis, years. Median (range)	10.3 (0.4–18.2)
Diagnosis	
Small vessel cPACNS	48 (33%)
Angiography positive, non-progressive cPACNS	22 (15%)
Angiography positive, progressive cPACNS	8 (6%)
Antibody-mediated IBrainD	26 (18%)
Anti-NMDA receptor encephalitis	13 (9%)
Other antibody-mediated	13 (9%)
Secondary IBrainD	12 (8%)
Secondary CNS Vasculitis	10 (7%)
Demyelinating	4 (3%)
Granulomatosis IBrainD	2 (1%)
Moyamoya-like	1 (1%)
T-cell mediated IBrainD	2 (1%)
IBrainD NYD	10 (7%)
Time to diagnosis, months. Median (range)	1.2 (0–150)

Legend: *Acronyms*: *CNS* Central nervous system, *cPACNS* Childhood primary angiitis of the central nervous system, *IBrainD* Inflammatory brain disease, *NYD* Not yet determined

### Clinical presentation

The most frequently reported symptoms at diagnosis were; cognitive/behavioural dysfunction 91 (63%) patients, seizures 65 (45%), stroke 64 patients (44%). The median disease activity score at diagnosis was high at 8.0 (range = 0–10), captured in 116 patients (80%). Neurologic function 1 year after diagnosis was measured in 106 patients; 43 (41%) poor outcome and 63 good outcome (59%). Clinical phenotypes and outcomes of the patient population are summarized in Table 2.

**Table 2:** Clinical phenotypes and outcomes for 145 pediatric patients with inflammatory brain disease

Clinical phenotypes at diagnosis	
Presenting symptoms	
Cognitive Dysfunction	91 (63%)
Seizures	65 (45%)
Hemiparesis	64 (44%)
Disease activity	
Number of patients with disease activity measurements	116 (80%)
Median (Range)	8.0 (0–10)
Estimated damage	
Number of patients with estimated damage measurements	98 (68%)
Median (Range)	1.0 (0–8)
Poor health-related quality of life	
Child’s self-reported	53% (18/34)
Parent’s proxy-reported	49% (19/39)
Outcomes	
Survival	144/145 (99%)
Neurologic function at 1 year	
Number of patients with neurologic function measurements	106 (73%)
Good	63/106 (59%)
Poor	43/106 (41%)
Poor health-related quality of life at 1 year	
Child’s self-reported	34% (16/47)
Parent’s proxy-reported	35% (17/48)

Legend: Disease activity and estimated damage were measured using the physician global assessment (PGA). The PGA is measured on a continuous scale from 0 to 10, with 0 representing no disease activity/damage, and 10 representing severe disease activity/damage. Neurologic function was measured using the pediatric stroke outcome measure (PSOM), with the presence of significant dysfunction in any of the 5 subdomains (right sensorimotor, left sensorimotor, language production, language comprehension and cognitive/behavioural deficit) indicating poor neurologic function

### Health related quality of life

A total of 145 patients completed at least one PedsQL questionnaire; at time of diagnosis, 34 child self reports and 39 parent proxy reports were completed. At 1 year after diagnosis 47 child self reports and 48 parent proxy reports were completed. A total of 98 patients completed a PedsQL questionnaire at more than one time point. Reasons for inability to complete the PedsQL were language limitations, poor medical status, lack of request by contributing physicians, or death.

The mean PedsQL scores at diagnosis were below the threshold reflecting poor HRQoL for both the child’s self-reported (mean 68.4 (range = 20.7–98.9)) and the parent proxy-reported (mean 63.4 (range = 13.0–98.9)). A total of 73 PedsQL questionnaires were completed at diagnosis: 34 child’s self-report and 39 parent’s proxy-report. Poor HRQoL at diagnosis was reported in 53% (18/34) of children, defined by PedsQL total scores less than or equal to 70, and 48% (19/39) of parents, defined by PedsQL scores of less than or equal to 65.

Mean PedsQL scores trended upward 1 year after diagnosis. A total of 95 PedsQL questionnaires were completed at 1 year after diagnosis; 47 child’s self-reports and 48 parent’s proxy-reports. The mean child’s self-reported PedsQL score 1 year after diagnosis was 72.5 (range = 6.3–98.9), while the mean parent proxy-reported PedsQL score was 66.7 (range = 14.1–95.65). A total of 34% (16/47) of children and 35% (17/48) of parents reported poor HRQoL 1 year after diagnosis.

HRQoL at diagnosis and at 1 year after diagnosis are described in Table 2.

### HRQoL subdomain functioning from parent and child perspectives at time-of-diagnosis

**Fig. 1:**
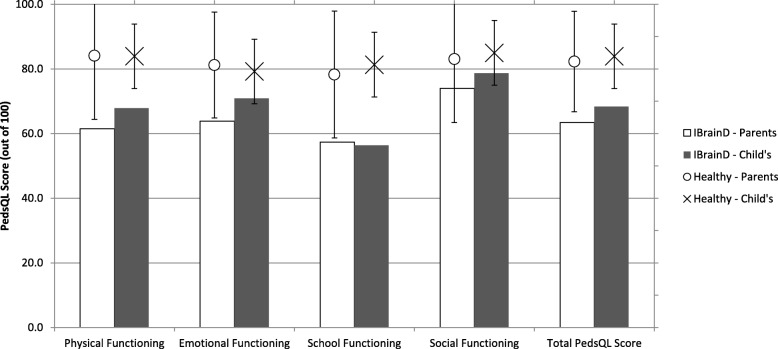
Health-related quality of life in children newly diagnosed with inflammatory brain disease, from the child’s perspective and the parent’s perspective. Legend: *n* = 34 for child’s self-reported PedsQL scores, and *n* = 39 for parent’s proxy-reported PedsQL scores. Reported values are mean baseline subdomain and overall PedsQL scores, out of 100, with larger scores representing improved quality of life

The mean child self-reported PedsQL subdomain score at diagnosis were; 67.9 (range = 9.4–100.0) physical functioning, 70.9 (range = 35.0–100.0) emotional functioning, 56.3 (range = 0.0–100.0 school functioning, and 78.7 (range = 0.0–100.0) social functioning. The mean parent proxy-reported PedsQL subdomain scores at diagnosis were; 61.4 (range = 0.0–100.0) physical functioning, 63.8 (range = 20.0–100.0) emotional functioning, 57.4 (range = 0.0–100.0) school functioning, and 74.0 (range = 0.0, 100.0) social functioning (see Fig. 1).

### Predictors of impaired HRQoL

The impact of patient characteristics and presenting clinical features on trajectories of HRQoL is summarized in Table 3, with increased risk of impaired HRQoL reflected by more negative parameter estimates.

**Table 3:** Impact of baseline patient characteristics and presenting clinical features on trajectories of health-related quality of life (HRQoL) over time

	Child self-reported HRQoL Parameter estimate β (SE^b^)	*P* value	Parent proxy-reported HRQoL Parameter estimate β (SE^b^)	*P* value
Predictors for impaired overall HRQoL trajectories over time				
Female Gender (vs. Male)	−7.6 (4.6)	0.10	−3.8 (4.4)	0.39
Seizures	−15.6 (4.6)^a^	< 0.01	−11.5 (4.4)	< 0.01
Cognitive Dysfunction	− 15.0 (4.8)^a^	< 0.01	−15.2 (4.6)	< 0.01
Hemiparesis	−2.9 (4.6)	0.53	−2.5 (4.4)	0.58
Predictors for impaired physical functioning trajectories over time				
Female Gender (vs. Male)	−8.9 (5.6)	0.12	−7.8 (5.9)	0.19
Seizures	−13.4 (5.7)	0.02	−9.9 (6.0)	0.10
Cognitive Dysfunction	−14.4 (5.9)	0.02	−11.4 (6.2)	0.07
Hemiparesis	−7.3 (5.7)	0.20	−1.7 (5.9)	0.77
Predictors for impaired psychosocial functioning trajectories over time				
Female Gender (vs. Male)	−7.1 (4.7)	0.13	−1.6 (4.3)	0.71
Seizures	−16.2 (4.7)	< 0.01	−12.4 (4.3)	< 0.01
Cognitive Dysfunction	−15.3 (4.9)^a^	< 0.01	−17.7 (4.4)^a^	< 0.01
Hemiparesis	−0.1 (4.7)	0.98	−0.1 (4.7)	0.44

Legend: Health-related quality of life (HRQoL) was measured using PedsQL 4.0 Generic Core Scales. Child-reported HRQoL was measured using the child self-reporting PedsQL questionnaire. PedsQL scores are between 0 and 100, with higher values representing better HRQoL. Negative changes in HRQoL associated with a clinical phenotype therefore represent risk factors for impaired HRQoL. 464 child self-reported PedsQL questionnaires were completed and used in this analysis. Each parameter estimate was derived from a separate, univariable linear regression model, adjusted for time, evaluating the impact of the predictor alongside time on HRQoL. Regression models were adjusted for repeated measures with an autoregressive covariance structure to establish whether these relationships changed statistically significantly with time

*n* = 145 patients

^a^asterisk indicates that the HRQoL impairment associated with the presence of the clinical variable at diagnosis is time-dependent, with the gap in HRQoL between patients with the clinical variable at diagnosis and patients who do not have the clinical variable at diagnosis decreasing with increasing time. This was only assessed in clinical parameters with statistically significant parameter estimates

^b^*SE* Standard error

Children with seizures at diagnosis had an increased risk of poor HRQoL, with dramatic impairments in both physical and psychosocial functioning subdomains. Child’s self-reported total PedsQL score at time-of-diagnosis was 15.6 points lower (*p* < 0.01) in children who had seizures at diagnosis, while the parent’s proxy-reported total PedsQL score at diagnosis was 11.5 points lower (p < 0.01), when compared to patients without seizures. PedsQL physical subdomain score at time-of-diagnosis was lower by 13.4 points (p < 0.01) in the child self-report in patients with seizures at diagnosis, and 9.9 points lower at time-of-diagnosis in the parent proxy-report, though not statistically significant (*p* = 0.10). Compared to patients without seizures at diagnosis, the child self-reported psychosocial subdomain PedsQL score at time-of-diagnosis was lower by 16.2 points (p < 0.01) in patients with seizures at diagnosis, and the parent proxy-reported psychosocial subdomain PedsQL score at diagnosis was lower by 12.4 points (p < 0.01). All reported values are parameter estimates at time-of-diagnosis.

Presenting with cognitive (and/or behavioural) dysfunction was a significant risk factor for impaired functioning across all subdomains. When compared to patients whithout cognitive dysfunction, the child self-reported total PedsQL in patients presenting with cognitive dysfunction was 15.0 points lower (p < 0.01) at time-of-diagnosis, the PedsQL physical subdomain score was 14.4 points lower (p < 0.01), and PedsQL psychosocial score was 15.3 points lower (p < 0.01). In the parent proxy-reports, cognitive dysfunction was associated with a decrease in the total PedsQL at time-of-diagnosis of 15.2 points (p < 0.01), and decrease in the psychosocial PedsQL at time-of-diagnosis of 17.7 points (p < 0.01). Cognitive dysfunction trended with a physical subdomain score at time-of-diagnosis 11.4 points lower than for those without cognitive dysfunction (*p* = 0.07). All reported values are parameter estimates at time-of-diagnosis, and are presented in Table 3.

Hemiparesis at diagnosis was not found to be a risk factor for impaired HRQoL.Male and female pediatric IBrainD patients did not have statistically different perceptions of physical or psychosocial functioning.

### Trajectories of HRQoL

Overall HRQoL, physical functioning, and psychosocial functioning were found to improve with time for all pediatric IBrainD patients, reflected by improving PedsQL scores with increasing log-transformed time from diagnosis. The relative improvement in HRQoL varied according to presenting features.

From the child’s perspective, improvement in HRQoL over time was seen in pediatric patients presenting with seizures or with cognitive/behavioural dysfunction relative to patients without these presenting features. This improvement was not observed from the parent’s viewpoint. Regression models adjusted for repeated measures showed that the overall HRQoL impairment for pediatric IBrainD patients presenting with seizures decreased with time (β = 2.7, SE =1.4, *p* = 0.05). From a separate model completed using the same methodology, patients with cognitive/behavioural dysfunction at diagnosis were found to self-report decreased overall HRQoL impairment (β = 3.5, SE =1.5, *p* = 0.02), as well as decreased impairment in psychosocial functioning over time (β = 3.6, SE =3.9, p = 0.02), when compared to patients without cognitive/behavioural dysfunction at diagnosis. Parent-perceived impairment in psychosocial functioning was found to decrease with log-transformed time for patients with cognitive dysfunction at diagnosis (β = 8.0, SE =3.9, *p* = 0.04).

By contrast, according to children’s self-reports, there was no improvement in physical functioning in patients presenting with either seizures or cognitive dysfunction compared to IBrainD patients who presented without these clinical features. Impairments in psychosocial functioning were not seen to decrease with time in patients presenting with seizures, relative to IBrainD patients without these clinical features. Parents did not perceive a decrease in impairment in overall HRQoL with increasing log-transformed time, for patients with seizures or cognitive dysfunction at diagnosis, compared to reports from parents of IBrainD patients without these clinical features. Parents also did not perceive decreased impairment in psychosocial functioning with increased log-transformed time for patients with seizures at diagnosis compared to patients without seizures at diagnosis.

### Continuous variables with associations with HRQoL

Improvement of HRQoL, reflected by improved PedsQL scores, was associated with older age at diagnosis, increased time between presentation and diagnosis, lower estimated damage at diagnosis, and higher estimated disease activity at diagnosis. Only the association between improvement of HRQoL and higher initial disease activity was statistically significant (β = 8.0, SE =3.9, p = 0.04). These trends were not found to significantly change with time.

## Discussion

This is the first study to systematically evaluate the impact of pediatric IBrainD on both overall HRQoL and its various subdomains. HRQoL is a multi-dimensional measurement used to estimate burden of disease. In the context of pediatric IBrainD, HRQoL will be critical in ensuring effective dialogue with affected children and families, and shaping future rehabilitation efforts. In IBrainD, the various subdomains of HRQoL are significantly impacted, with the greatest burden of disease in children presenting with cognitive/behavioural dysfunction or seizures.

HRQoL in pediatric patients with IBrainD is significantly impaired, with mean PedsQL scores at diagnosis consistently dramatically lower than mean PedsQL scores in healthy children, as reported by Varni et al., reflecting significant burden of disease [9]. Based on greater than 5000 child self-reports, Varni et al. determined the mean total PedsQL score in healthy children to be 83.9 (SD = 12.5) [9]. This is almost 25% higher than the mean PedsQL score for children with IBrainD. School functioning was found to be the most affected PedsQL subdomain at baseline; Varni et al.’s healthy pediatric population had a mean PedsQL score almost 1.5 times greater than children newly diagnosed with IBrainD [9]. Social functioning was found to be the least affected of all the subdomains, with healthy children scoring 8% higher than pediatric IBrainD patients [9]. The mean physical and emotional subdomain PedsQL scores for healthy children as reported by Varni et al. were 24 and 12% higher, respectively, than those calculated for pediatric patients newly diagnosed with IBrainD [9]. This follows a similar trend to pediatric patients with MS; in a study by MacAllister, 46% of pediatric patients with MS self-reported physical impairment, 42% academic impairment, 28% emotional impairment, and only 14% social impairment at baseline [16]. A study by Russell et al. following adolescents with sports-related concussions found the greatest impairments at clinical presentation to be in the physical and cognitive subdomains [17]. School functioning for patients with IBrainD is especially affected at time of diagnosis, potentially due to extensive school absences owing to hospitalization. Consequently, attention should be given to determine if poor school functioning in children with IBrainD recovers with time or if additional cognitive rehabilitation services are required.

Children presenting with seizures or cognitive/behavioural dysfunction have an especially high risk of long-term impairment of HRQoL, with reduced mean PedsQL scores across all subdomains of HRQoL. These findings were consistent with findings in a study of children following acquired brain injury, where post-injury cognition/learning impairment was associated with worse overall HRQoL, particularly in the psychosocial domain, and post-injury behavioural impairment was moderately associated with reduced psychosocial functioning [18]. It may be postulated that seizures and cognitive/behavioural dysfunction fit within a phenotypic cluster associated with certain conditions previously identified to have poorer prognoses; for example, concentration difficulties, cognitive dysfunction, and mood/personality changes were found to be more common in the APP-cPACNS population, whereas hemiparesis was found to occur more frequently in the APNP-cPACNS cohort and seizures were common in SV-cPACNS [1, 19]. Alternatively, the prognosis of pediatric IBrainD patients with hemiparesis may be more favorable as a result of high availability and routine utilization of targeted physical rehabilitation efforts for children with physical impairment. In comparison, rehabilitation efforts and social services targeting children with seizures or pathological cognitive and/or behavioural dysfunction is typically limited or absent. Though mean HRQoL tended to increase for all patients, as consistent with the ‘response shift’ process [20], the impairments in functioning associated with seizures were found to persist through time, and the diminished quality of life associated with behavioural/cognitive dysfunction was found to recover only very slowly. More rigorous and targeted attention and intervention should be initiated at diagnosis for this group of patients.

There are a number of limitations to this study. One limitation concerns generalizability to children with IBrainD presenting with severe disability, as acutely hospitalized children with significant impairment were often unable to complete the PedsQL questionnaires. As a result, the true impact was likely underestimated in this study. Specifically, the self-reported pediatric HRQoL for children with cognitive dysfunction at diagnosis was likely significantly poorer than reported, as this population likely encountered significant barriers understanding and completing the PedsQL questionnaires. In addition, while the results are applicable to IBrainD as a whole, they may not be generalizable to all distinct disease subtypes, explaining the variability of patient presentations. For example, while some IBrainDs have abrupt and severe symptom onset, others progress more insidiously, often resulting in a prolonged time to diagnosis. Separation of these disease subtypes may be considered for future studies. Finally, the BrainWorks study was a largely unfunded, international, multi-center study, and accordingly, adherence to study guidelines was variable, resulting in missing and incomplete data. However, as this study is the first to evaluate HRQoL in pediatric IBrainD patients using validated study instruments, it is the only information to date pertaining to the impact of any of the pediatric IBrainDs on perceptions of functioning and disability. Analysis of this data provides valuable initial documentation of the burden of IBrainD.

## Conclusion

This study is the first to evaluate the burden of pediatric IBrainD on the HRQoL. While differences of opinion exist regarding whether the greatest value from the PedsQL tool is ascertained through the objective components, such as observation of activity and interaction by parents via the parent-proxy report, or via the subjective component that allows the child to self-report their own experience of impairment, it is agreed upon that the PedsQL can provide valuable insight into the patient experience of illness [10]. IBrainD was found to result in significant HRQoL impairment, with the greatest disabilities in the school functioning and physical functioning subdomains at diagnosis. Children presenting with seizures and cognitive dysfunction were found to have a higher risk of impairment, both at diagnosis and in the future, mandating improvements in rehabilitation and social service strategies targeting these patients. While rehabilitation programs targeting physical deficits are currently robust, there is a clear unmet demand for rehabilitation for children with pathologic cognitive or behavioral dysfunction, targeting psychosocial recovery. Future work should strive to improve the prognosis for children with IBrainD, especially those presenting with risk factors for increased burden of disease. Identification of patients with increased risk of poor HRQoL should be followed by the provision of targeted rehabilitation, which aims to improve functioning in the subdomains predicted to have weak recovery.

## Abbreviations

APNP-cPACNS: Angiography-positive non-progressive childhood primary angiitis of the CNS
APP-cPACNS: Angiography-positive progressive childhood primary angiitis of the CNS
cPACNS: Childhood primary angiitis of the CNS
HRQoL: Health-related quality of life
IBrainD: Inflammatory brain disease
MS: Multiple sclerosis
NMDA: N-methyl-D-aspartate
PedsQL: Pediatric Quality of Life Inventory Version 4.0
PedVas: Pediatric Vasculitis Initiative
PGA: Physician global assessment
PSOM: Pediatric stroke outcome measure
SD: Standard deviation
SV-cPACNS: Small vessel childhood primary angiitis of the CNS
